# Awareness and Practices Regarding Leptospirosis in Andaman and Nicobar Islands, Union Territory of India

**DOI:** 10.7759/cureus.44305

**Published:** 2023-08-29

**Authors:** Aanchal Anand, Pragya Sharma, Ajay Raj Sethuraman, Sabaa Begum, Vanni Anand

**Affiliations:** 1 Community and Family Medicine, Andaman and Nicobar Islands Institute of Medical Sciences (ANIIMS), Port Blair, IND; 2 Community Medicine, Maulana Azad Medical College, New Delhi, IND; 3 Community Medicine, Andaman and Nicobar Islands Institute of Medical Sciences (ANIIMS), Port Blair, IND

**Keywords:** andaman and nicobar, epidemiology, practices, awareness, leptospirosis

## Abstract

Background

Leptospirosis is a worldwide prevalent zoonotic infection and re-emerging disease caused by the bacterium of genus *Leptospira* transmitted by infected animals in the environment. Andaman and Nicobar Islands has one of the highest incidence rates of leptospirosis in India with a seroprevalence of 52.7%. This study examined the knowledge, attitudes, and practices (KAP) regarding leptospirosis among the urban and rural populations of the Union Territory (UT) of India.

Aim and objective

This study aims to assess the knowledge, attitude, and practices regarding leptospirosis in a rural and an urban community of Andaman and Nicobar Islands.

Materials and methods

This community-based cross-sectional study was conducted at rural and urban field practice areas under the aegis of the Department of Community Medicine, Andaman and Nicobar Islands Institute of Medical Sciences (ANIIMS), Port Blair, for a period of three months. A semi-structured interview schedule was administered to 450 participants in community settings selected from a list of households obtained from the municipal council using a systematic random sampling method. All interviews were conducted face-to-face by the investigators to collect data on the sociodemographic variables of the study participants and their knowledge, attitudes, and practices regarding the disease. The data was analyzed using Statistical Package for the Social Sciences (SPSS) version 27.0 (IBM SPSS Statistics, Armonk, NY, USA).

Results

The knowledge and attitude regarding leptospirosis and henceforth the likelihood of individuals using preventive practices were evaluated. The majority of respondents (97%) had previously heard about leptospirosis; however, specific knowledge regarding its risk factors, causative agent, and complications was limited among the participants. Although more than 90% of them had a favorable attitude toward seeking treatment for the disease and adopting specific preventive measures and general hygiene, they did not practice these hygiene habits nor used self-protective equipment in their day-to-day lives. Less than 50% of participants wore protective clothing, boots, and gloves while cleaning cattle sheds, and only 40% of them used rodenticides despite the presence of rodents in their houses. Most of the owners (60%) had not vaccinated their pets. We also found a significant association between the male gender and urban residence with a good knowledge score (p=0.05).

Conclusion

Despite having knowledge of and a positive attitude toward the disease, the participants did not practice preventive measures. We recommend that health interventions in endemic communities should focus on the implementation of protective measures by raising awareness in the susceptible population on multiple dimensions of leptospirosis in order to attain and ensure better compliance.

## Introduction

Andaman and Nicobar Islands, being a part of a tropical belt, leaves its population highly vulnerable to contracting leptospirosis, a zoonotic disease favored by humid climate, heavy rainfall, and occasional flooding and characterized by multi-organ failure as one of its complications [1-3]. Despite its high prevalence, people are often negligent about this disease and its preventive measures, leading to significant morbidity and mortality in the Union Territory (UT) of India [4].

Hence, this study aimed to assess the knowledge, attitudes, and practices (KAP) regarding leptospirosis in a rural and an urban community of Andaman and Nicobar Islands in order to identify interventions required in the community for the prevention and early diagnosis of leptospirosis to reduce disease burden.

## Materials and methods

Study design

This study is a community-based cross-sectional study.

Study area

The study was conducted among both rural and urban field practice areas under the Department of Community Medicine, Andaman and Nicobar Islands Institute of Medical Sciences (ANIIMS), Port Blair. The rural population was taken from Chouldhari village located in South Andaman district with a total population of 5,009 as per the records maintained at the Health and Wellness Centre, Chouldhari, and the urban population was chosen from Garacharma area in South Andaman district with a total population of 14,419 as per the records maintained at the Health and Wellness Centre, Garacharma.

Study period

The study was conducted from January to March 2023.

Study population

The study population consisted of adults (18 years and above) residing in both study areas.

Inclusion Criteria

All males and females 18 years and above who had been residing in the study area for at least one year.

Exclusion Criteria

All those diagnosed with leptospirosis by any healthcare facility or had any family member treated for the disease.

Study tool

A pre-tested, semi-structured interview schedule was administered to all the participants after obtaining their written consent. The pre-testing was carried out in Ferrargunj and Haddo, which are rural and urban areas, respectively.

The interview schedule comprised four sections: sociodemographic characteristics and knowledge, attitudes, and practices regarding leptospirosis. The sociodemographic section gathered details such as name, age, gender, address, educational qualifications, occupation, and socioeconomic class. The knowledge section comprised a set of questions addressing the epidemiology, transmission, clinical features, diagnostic methods, outcomes, prevention, and treatments available for leptospirosis. The attitude of the population regarding the disease was assessed thoroughly by questions on vaccines for domestic animals and chemoprophylaxis and adopting preventive measures to avoid infection and transmission of the disease. Practices regarding prevention were assessed through questions based on adherence to preventive measures, frequency of handwashing, eating habits, prevention of food and water contamination, protection of domesticated animals, and use of personal protective equipment. Multiple responses were allowed in suitable questions to gather detailed perceptions of the study participants regarding the disease.

Sample size

For the calculation of sample size, the following formula was used: N = Z^2^_(1-α/2)_ * P(1-P)/d^2^, where N is the sample size, Z _(1- α/2)_ is 1.96 value of the standard normal variate corresponding to the level of significance α of 0.5, d is the specified precision on either side of the mean, and P is prevalence.

The sample size was calculated assuming a 95% confidence level, a 5% margin of absolute error, and a 50% proportion for prevalence. Since there has been no similar study conducted in Andamans previously, we have taken the prevalence to be 50% [5]. Taking into account a 10% non-response rate, a total of 450 subjects were enrolled in the study.

Methodology

The list of households was taken from Port Blair Municipal Council for the designated rural and urban areas, and the total number of eligible households based on inclusion criteria was short-listed. Systematic random sampling was used to select the households out of a total number of eligible households. The first household was selected by simple random sampling using a random number generator in Microsoft Excel software (Microsoft Corporation, Redmond, WA, USA). After selecting the household, one study participant was selected by a simple random sampling technique using a random number method and enrolled in the study. If a particular house was found locked on three consecutive visits, then that household was dropped from the study and the next eligible household was chosen. All interviews were conducted at participants’ homes through face-to-face interaction with the investigator to collect data on sociodemographic details and knowledge, attitudes, and practices related to leptospirosis.

Ethical considerations

The study protocol was approved by the Institutional Scientific Research Committee (ISRC) and the Institutional Ethics Committee, ANIIMS, Port Blair. Written informed consent was obtained from the study participants after explaining to them the nature and purpose of the study.

Data analysis

Data was entered in Microsoft Excel (Microsoft Corporation, Redmond, WA, USA), and analysis was done using Statistical Package for the Social Sciences (SPSS) version 27.0 (IBM SPSS Statistics, Armonk, NY, USA). Quantitative variables were expressed as means and standard deviations. Categorical variables were expressed as frequencies and percentages. The chi-square test was used to test differences in proportions. A two-tailed p-value of <0.05 was considered statistically significant.

## Results

Sociodemographic characteristics

The age of the study participants ranged from 18 to 80 years, and their mean age was 43.36±5.7 years. There was nearly an equal proportion of male (51.3%) and female (48.7%) participants in our study. Most of the participants were either graduates (22.2%) or had completed middle school (22%), followed by high school (18%). More than two-thirds of the participants belonged to nuclear families (72.7%) comprising parents and children (Table 1).

**Table 1:** Distribution according to demographic variables INR: Indian rupee

Age (years)	Frequency	Percentage	
18-30	115		25.6%
31-40	91		20.2%
41-50	103		22.9%
51-60	80		17.8%
61-70	40		8.8%
>70	21		4.7%
Gender			
Male	231		51.3%
Female	219		48.7%
Marital status			
Unmarried	82		18.2%
Married	354		78.7%
Widow	14		3.1%
Residence			
Urban	238		52.9%
Rural	212		47.1%
Religion			
Hindu	386		85.8%
Muslim	29		6.4%
Christian	35		7.8%
Education			
Illiterate	33		7.3%
Primary school	76		16.9%
Middle school	99		22%
High school	81		18%
Intermediate	61		13.6%
Graduate and above	100		22.2%
Family type			
Nuclear	327		72.7%
Joint family	94		20.9%
Three generations	29		6.4%
Number of family members			
1-2	54		12%
3-5	323		71.8%
6-9	61		13.6%
≥10	12		2.6%
Total family income per month (INR)			
≥52,734	79		17.6%
26,355-52,733	112		24.9%
19,759-26,354	83		18.4%
13,161-19,758	79		17.6%
7,887-13,160	75		16.7%
2,641-7,886	18		4%
≤2,640	4		0.8%

Knowledge

Among the total participants, almost 97% of the participants had heard about leptospirosis, while 3.1% were completely unaware of the disease. While 24% of the people considered bacteria to be the causative organism, followed by viruses (19.6%), 2.9% of the participants thought rats, rodents, and mosquitoes as the causative organism, while 37.1% of the participants were not aware of the cause of the disease. Animals (65.3%) were mostly considered to be the mode of transmission, followed by mosquitoes (48.9%), contaminated water (39.1%), and food (36%). Nearly 61% of the people thought walking barefoot was the most significant risk factor, followed by contact with contaminated animal carcasses (48.9%) and water bodies (47.1%), while only four (0.9%) participants specifically mentioned contact with infected rat’s urine as a risk factor. More than 75% of the participants considered floods to be the most potential natural calamity responsible for disease transmission.

Fever (74.7%), headache (54.7%), vomiting (43.8%), muscle pain (43.3%), weakness (40.4%), and redness of the eyes (21.3%) were thought to be the most common symptoms, and jaundice (42.9%) and liver damage (26.9%) as the most common complications of leptospirosis. Blood tests (69.1%) followed by urine examination (13.6%) were most commonly thought of as the methods of diagnosis, but 1.1% of the participants were also of the opinion that this disease cannot be diagnosed. While 72.4% of the people considered leptospirosis to be a fatal disease, about 2.5% disagreed, and the rest were unaware of its outcome.

More than 86% of the participants were of the opinion that leptospirosis can be prevented with the use of protective equipment, proper hand hygiene, avoiding direct contact with animals and flooded areas, and anti-rodent measures, but only 3.1% and 10.2% of them were aware of the availability of chemoprophylaxis and vaccines for domestic animals against leptospirosis, respectively (Table 2).

**Table 2:** Assessment of knowledge related to leptospirosis

Knowledge assessment	Frequency	Percentage
Do you know about leptospirosis/Andaman fever?		
Yes	436	96.9%
No	14	3.1%
What do you think is the causative agent for leptospirosis?		
Bacteria	108	24%
Fungi	5	1.1%
Virus	88	19.6%
Parasite	55	12.2%
Insects, rats, rodents, and mosquitoes	13	2.9%
I don’t know	167	37.1%
Do you know how leptospirosis is transmitted?		
Animals	294	65.3%
Mosquitoes	220	48.9%
Houseflies	98	21.8%
Blood	64	14.2%
Contaminated food	162	36%
Contaminated water/drinks	176	39.1%
Respiratory droplets/nuclei	8	1.8%
Direct contact with an infected individual	22	4.9%
Do you know what the risk factors for leptospirosis are?		
Handling animals with bare hands	206	45.8%
Contact with contaminated bodies of dead animals	220	48.9%
Walking barefoot	274	60.9%
Contact with flood/water bodies	212	47.1%
Open sewers	151	33.6%
Direct contact with the urine of an infected animal	4	0.9%
Which of the following natural calamities is a risk factor for leptospirosis?		
Draught	57	12.7%
Flood	354	78.7%
Avalanche	1	0.2%
Landslide	54	12%
Cyclone	93	20.7%
Rain and tsunami	12	2.7%
I don’t know	12	2.7%
Do you know the symptoms of leptospirosis?		
Fever	336	74.7%
Headache	246	54.7%
Nausea	133	29.6%
Vomiting	197	43.8%
Redness of the eyes	96	21.3%
Muscle pain	195	43.3%
Chills	149	33.1%
Weakness	182	40.4%
Diarrhea	73	16.2%
All of these	96	21.3%
Pruritus	1	0.2%
Rashes	1	0.2%
No	7	1.6%
How can this disease be diagnosed?		
I don’t know	113	25.1%
It can’t be diagnosed	5	1.1%
Blood test	311	69.1%
Chest X-ray	12	2.7%
Urine examination	61	13.6%
Do you know about the complications of leptospirosis?		
Jaundice	193	42.9%
Cancer	5	1.1%
Liver damage	121	26.9%
Anemia	86	19.1%
Kidney failure	92	20.4%
Heart failure	49	10.9%
Breathing difficulty	104	23.1%
I don’t know	158	35.1%
Any other	6	1.3%
Can leptospirosis cause death?		
Yes	326	72.4%
No	11	2.4%
I don’t know	99	22%
Can the spread of leptospirosis be prevented?		
Yes	390	86.7%
No	45	10%
I don’t know	1	0.2%
Which of the following are measures to prevent leptospirosis?		
Anti-rodent measures	113	25.1%
Avoiding contact with flooded areas	139	30.9%
Avoiding direct contact with animals	146	32.4%
Proper hand hygiene measures	168	37.3%
Using protective equipment/clothing	56	12.4%
All of these	174	38.7%
Any other	1	0.2%
Are you aware of chemoprophylaxis available for leptospirosis?		
Yes	14	3.11%
No	436	96.89%
Are you aware of vaccines for animals available to prevent leptospirosis?		
Yes	46	10.2%
I don’t know	404	89.8%

Attitude

More than 90% of the participants had a positive attitude toward treatment seeking, adopting specific preventive measures against leptospirosis, and general hygiene measures such as handwashing. However, 4.4% of the population did not consider it necessary to seek medical help if one develops symptoms of leptospirosis, while 10.22% and 3.1% of people, respectively, refused the need to take preventive measures such as vaccines for domestic animals and chemoprophylaxis against the same. A small segment of the study subjects did not deem the following measures as necessary to prevent leptospirosis: making the environment free of rodents (12%), wearing protective equipment while cleaning cattle sheds/kennels/sty, vaccinating their domesticated animals and disposing of the remains of animals properly (1.3%), covering household drains (0.7%), and cleaning them frequently (1.3%). The remaining participants had a favorable attitude toward all these preventive measures (Table 3).

**Table 3:** Attitude toward leptospirosis

Attitude				
	Yes		No	
	Frequency	Percentage	Frequency	Percentage
Do you think it is necessary to seek medical help if one has symptoms of leptospirosis?	430	95.6%	20	4.4%
Do you think we need to take preventive measures against leptospirosis?	418	92.9%	32	7.1%
Do you think it is necessary to take chemoprophylaxis or vaccines for domestic animals to prevent leptospirosis?	403	89.6%	47	10.4%
Do you think making your environment free from rodents is important?	396	88%	54	12%
Do you think cleaning the cattle sheds/pigsty/kennels daily is important?	446	99.1%	4	0.9%
Do you think it is important to wear protective equipment (e.g., gloves and boots) while cleaning cattle sheds/pigsty/kennels?	444	98.7%	6	1.3%
Do you think vaccinating pet dogs/cattle/pigs/goats is important?	444	98.7%	6	1.3%
Do you think it is important to dispose of dead animal’s remains?	444	98.7%	6	1.3%
Do you think it is important to ensure that the pond or lake in your surroundings is not contaminated?	448	99.6%	2	0.4%
Do you think wearing footwear while walking in the fields or kutcha roads is important?	450	100%	0	0%
Do you think it is important to wash your feet after they come in contact with soil?	449	99.8%	1	0.2%
Do you think maintaining hand hygiene practices is important?	450	100%	0	0%
Do you think it is important to wash vegetables before eating them raw?	449	99.8%	1	0.2%
Do you think it is important to store leftover foods by either covering or refrigerating them?	448	99.6%	2	0.4%
Do you think it is important to store water in covered containers?	450	100%	0	0%
Do you think it is important to dispose of kitchen waste properly?	450	100%	0	0%
Do you think it is important to dispose of animal refuse properly?	448	99.6%	2	0.4%
Do you think it is important to cover household drains?	444	98.7%	6	1.3%
Do you think it is important to clean the household drains frequently?	447	99.3%	3	0.7%

Practices**

Although 88% of the participants had a positive attitude toward making their environment rodent-free, only 40% of them used rodenticides in their homes despite the presence of rodents. Less than 50% of those owning a domestic animal had them vaccinated. While more than 65% of cattle owners cleaned the cattle shed/kennel/sty daily, among them only two-fifths wore boots and gloves while cleaning them.

While 57.1% of the people threw the leftover food or kitchen waste in a separate bin for wet waste, others preferred to throw them in a nearby drain (32.7%) or in an open area (9.6%). About 55% of the people interviewed disposed of their household waste daily, whereas 32.2% disposed biweekly, and 9.1% did it once weekly. More than 36% of the participants did not keep the drains in their vicinity covered. Around 41.6% of the participants cleaned the drains as required, while 12.2% of them never cleaned the drains (Table 4).

**Table 4:** Practices related to leptospirosis

Practices	Frequency	Percentage
Do you use rodenticides?		
Yes	176	39.1%
No	274	60.9%
Do you clean the cattle sheds/pigsty/kennel daily?		
Yes	71	15.8%
No	34	7.6%
Not applicable	345	76.7%
Do you wear gloves and boots while cleaning cattle sheds?		
Yes	43	9.6%
No	60	13.3%
Not applicable	347	77.1%
Are your cattle/pets vaccinated?		
Yes	50	11.1%
No	98	21.8%
Not applicable	302	67.1%
Do you wear footwear when you walk in fields/muddy areas?		
Yes	410	91.1%
No	40	8.9%
Do you wash your hands and feet when coming in contact with soil?		
Yes	439	97.6%
No	11	2.4%
What do you use to wash your hands and feet?		
Only water	89	19.8%
Water and soap	361	80.2%
What do you do before cooking vegetables?		
Wash them with water	115	25.6%
Neither wash nor boil the vegetable	35	7.8%
Both wash and boil the vegetable	300	66.7%
How do you store leftover foods?		
Close the lid of the food container	147	32.7%
Keep leftover foods in the refrigerator	303	67.1%
Throw away	1	0.2%
How do you dispose of leftover foods?		
Throw away in the open	43	9.6%
Throw in a nearby drain	143	31.8%
Throw in a separate garbage bin for wet waste	257	57.1%
Make compost	7	1.5%
How frequently do you dispose of household waste?		
Everyday	264	58.7%
Biweekly	145	32.2%
Once a week	41	9.1%
Do you keep the stored water in covered containers?		
Yes	441	98%
No	9	2%
Do you keep the drains in your vicinity covered?		
Yes	286	63.6%
No	164	36.4%
How frequently do you clean the drains?		
As and when required	187	41.6%
Occasionally	208	46.2%
Never	55	12.2%

Association between demographic factors and knowledge score

The demographic variables gender (chi-square=11.886, p=0.008) and residence (chi-square=35.823, p<0.001) are significantly associated with the knowledge score. No significant association between knowledge and age, marital status, religion, education, and family type was observed (p>0.05) (Table 5).

**Table 5:** Association between demographic factors and knowledge score *Statistically significant

		Knowledge score				Chi-square	p-value
		Low	Average	Good	Excellent		
Age	18-30	4	9	85	17	20.564	0.151
	31-40	1	16	65	9		
	41-50	3	10	77	13		
	51-60	5	3	65	7		
	61-70	0	6	27	7		
	>70	1	4	15	1		
Gender	Male	4	34	170	23	11.886	0.008*
	Female	10	14	164	31		
Marital status	Unmarried	3	5	60	14	5.341	0.501
	Married	11	42	262	39		
	Widow	0	1	12	1		
Residence	Urban	8	29	193	8	35.823	0.001*
	Rural	6	19	141	46		
Religion	Hindu	13	42	282	49	3.789	0.705
	Muslim	1	3	24	1		
	Christian	0	3	28	4		
Education	Illiterate	2	5	25	1	20.88	0.141
	Primary school	0	10	62	4		
	Middle school	3	12	73	11		
	High school	1	11	57	12		
	Intermediate	2	4	44	11		
	Graduate	6	6	73	15		
Family type	Nuclear	8	38	242	39	8.525	0.202
	Joint family	5	7	67	15		
	Three generations	1	3	25	0		

## Discussion

Although several serological studies to establish the association of leptospirosis with certain high-risk occupations have been carried out, studies to explore awareness and preventive practices are still lacking. Andaman and Nicobar Islands, being a highly endemic pocket, require robust data on existing awareness and practices for initiating effective control measures.

The present study demonstrated that 96.9% of the participants were aware of the disease, but they recognized it by the name of “Andaman fever.” Animals (65.3%) were mostly considered to be the mode of transmission, followed by mosquitoes (48.9%), contaminated water (39.1%), and food (36%). Similar findings were observed in a study conducted by Sukeri et al. among the rural and urban communities in Malaysia in 2018, in which the findings revealed that almost all the participants had heard of leptospirosis or rat urine disease but lacked basic knowledge regarding the disease [6]. This was in contrast to the findings of a cross-sectional study conducted in Tamil Nadu by Prabhu et al. among municipal workers, where it was found that only 18.9% of the workers had heard about leptospirosis, while most of the participants had false beliefs that leptospirosis can be transmitted by mosquitoes as well and can cause lung cancer in later stages [7]. The findings in our study are heartening as even the general community here is familiar with the disease against the municipal workers of Tamil Nadu who are at high risk for it.

Nearly 61% of the people thought walking barefoot was the most significant risk factor, followed by contact with contaminated animal carcasses (48.9%) and water bodies (47.1%) However, only 0.9% of the participants specifically mentioned infected rat urine as a risk factor for leptospirosis. This was in contrast with the findings of a cross-sectional study conducted by de Araújo et al. in the urban slums of Salvador, Brazil. where 72.7% of the participants were aware that leptospirosis is a disease spread by rats [8]. The difference in the findings can be attributed to the high literacy rate (99.2%) and health awareness among the Brazilian population [9].

Over 70% of the people were of the opinion that leptospirosis is a fatal disease. Similar conclusions were drawn from a cross-sectional study conducted by Ricardo et al. in 2017 among the residents of riverside settlements in Santa Fe, Argentina, where nearly 80% of the participants agreed that leptospirosis can cause death if not treated. More than 80% of the participants also considered leptospirosis to be a preventable disease in corroboration with 86% of the participants of our study [10].

More than 90% of the participants had a positive attitude toward treatment seeking, adopting specific preventive measures against leptospirosis, and general hygiene measures such as handwashing. Of the participants, 88% considered making their environment free of rodents as an effective method to prevent leptospirosis. This was consistent with the findings observed in a cross-sectional study conducted by Nair et al. in 2020 in Thiruvananthapuram, Kerala, where 85.63% of the respondents expressed a positive attitude toward keeping their houses rat-free [11].

Surprisingly, despite the majority of participants having knowledge and positive attitudes regarding the disease, only 40% of them practiced the use of rodenticides. This corroborated the findings of the study conducted in 2011 by Mohan and Chadee in Trinidad, in which 68.9% of the participants did not spend any money on rodent control [12]. Less than 50% of those owning domestic animals got them vaccinated. Although more than 65% of cattle owners cleaned the cattle shed/kennel/sty daily, only around 40% took preventive measures and wore boots and gloves while cleaning them. Similar findings were seen in a cross-sectional study conducted by Arulmozhi et al. in 2017 among the risk populations of South Chennai, in which 39% of the participants did not use any protective equipment while engaging in high-risk activities [13].

We found the demographic variables gender (chi-square=11.886, p=0.008) and residence (chi-square=35.823, p<0.001) to be significantly associated with the knowledge score. However, no significant association between knowledge and age, marital status, religion, education, and family type was observed (p>0.05). This was in contrast to the study conducted by Rathinam et al. from March 2017 to March 2018 in Madurai, in which none of the factors such as age, gender, and residence were found to be significantly associated with awareness of preventive measures against leptospirosis [14].

Limitations

This was a cross-sectional study in which the responses were self-reported and hence prone to recall bias, and also, the risk of social desirability is anticipated to be high. Moreover, the changes especially in attitude and practices could not be evaluated further as it was a cross-sectional study.

Strengths

Nevertheless, this study is the first of its kind to be conducted in the Union Territory with high endemicity and seroprevalence of leptospirosis. The study involves both urban and rural populations of the islands, providing us with a scope to assess a difference in sociodemographic determinants of the disease. Also, the findings of our study can be extrapolated to both urban and rural settings. It was conducted in community settings in the participants’ own households without the time constraints of a facility-based survey, enabling interactive and active participation, henceforth improving the quality of responses.

## Conclusions

A community-based cross-sectional study was conducted at rural and urban field practice areas under the aegis of the Department of Community Medicine, Andaman and Nicobar Islands Institute of Medical Sciences, Port Blair, in which 450 participants were enrolled. Around 52.89% of the participants in the study belonged to a rural area, while 47% of them were urban residents. Almost all the participants had heard about leptospirosis; however, when probed further about the specificities of the disease, such as modes of transmission, risk factors, complications, and outcome, the knowledge gap was revealed. Very few participants were aware of chemoprophylaxis and the availability of vaccines for domestic animals against leptospirosis. The majority of participants had a positive attitude toward adopting preventive measures in their day-to-day lives involving personal hygiene, environment hygiene, and kitchen hygiene. Although the participants had basic knowledge and positive attitudes toward its preventive measures, they failed to translate these into daily practices. The majority of them did not use rodenticides despite the presence of rodents in their home, and only half of those owning domestic animals got them vaccinated. Also, despite knowing that poor environmental hygiene increases the risk of the transmission of leptospirosis, nearly two-fifths of the participants threw away the garbage in nearby drains or in open areas, increasing the risk of the spread of leptospirosis infection.

The study provided evidence of failure to translate knowledge about the disease to enactment of preventive measures despite the high prevalence of leptospirosis in the UT. Thus, there is a need to organize awareness programs by public health experts in schools, colleges, and healthcare facilities ensuring the involvement of students, teachers, and healthcare workers, as well as veterinary and agricultural departments, to reinforce the implementation of preventive practices in day-to-day lives. Also, the implementation of these measures has to be periodically evaluated among the population by grassroots-level workers to assess the impact of awareness programs and municipal authorities to mitigate the environmental risk factors of the disease.
